# The role of condition on sexual selection in the seed bug *Lygaeus simulans*

**DOI:** 10.1002/ece3.70226

**Published:** 2024-09-04

**Authors:** Vicki L. Balfour, Mia K. Corliss, David M. Shuker

**Affiliations:** ^1^ School of Biology University of St Andrews St Andrews UK

**Keywords:** condition‐dependent, cryptic male choice, environmental stress, mate choice, mating failure, post‐copulatory sexual selection

## Abstract

Organism condition plays an important role in sexual selection. Sexual ornaments and displays can be condition‐dependent, reflecting either underlying genetic quality, experience of environmental stressors, or both. As such, the phenotypic expression of such traits, and the resulting patterns of mate choice acting on them, may be shaped by intrinsic genetic quality and the environment. Moreover, condition may also influence the choosing individual in mate choice, influencing their ability to invest in mate discrimination, or changing what traits of the chosen, including resources, are most preferred. Here we consider sexual selection and condition in the seed bug *Lygaeus simulans*, a species characterised by strong post‐copulatory sexual selection, but rather limited pre‐copulatory discrimination. We manipulated short‐term condition in both males and females by restricting access to water for 24 h. Water is particularly important in these bugs, given their feeding ecology and physiology. We found that water‐deprived males proved less likely to mate, while copulation duration with water‐deprived females was significantly reduced. Given the importance of copulation duration for the successful transfer of sperm by males to females, the data suggest cryptic male choice acting against water‐deprived females. These data add to those suggesting that cryptic male choice for fecund females plays an important role in sexual selection in this species. More generally, our results support the widespread importance of condition in terms of mating dynamics and sexual selection.

## Graphical abstract

Organism condition plays an important role in sexual selection. Here, we manipulated condition in the seed bug *Lygaeus simulans*, by restricting access to water 24 h prior to a mating opportunity. We found that water‐deprived males proved less likely to mate, while copulation duration with water‐deprived females was significantly reduced. Given the importance of copulation duration for the successful transfer of sperm by males to females, the data suggest cryptic male choice acting against water‐deprived females.

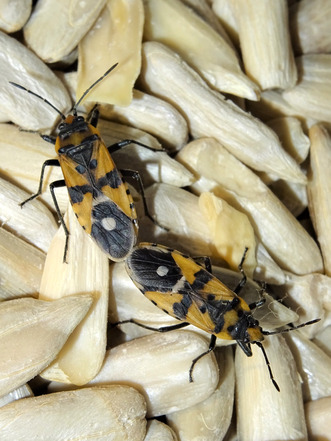

## 1 INTRODUCTION

The role of body condition has long been associated with the study of sexual selection. While Darwin emphasised mate choice and a ‘sense of the beautiful’ underlying mate preferences, he also noted that it is likely that successful males are also the most vigorous and healthy (Darwin, 1871). The role of condition has also been central to the modern study of sexual selection. An animal's condition will in part reflect its underlying genetic quality, where quality is usually taken to mean its overall quality in terms of components of naturally selected fitness (resistance to parasites, metabolic efficiency, ability to evade predators, efficient energy allocation strategies and so forth: Andersson, 1994; Hamilton & Zuk, 1982; see below). The extent to which loci across the genome contribute to these components of fitness are the extent to which ‘good genes’ exist across the genome. Key to understanding the role of good genes in sexual selection has been to work out if and how courtship displays or ornaments reflect or capture that underlying genetic condition (Rowe & Houle, 1996; Tomkins et al., 2004; Whitlock & Agrawal, 2009). Moreover, sexual selection favouring overall genetic condition can help purge genomes of deleterious alleles and positively influence population fitness (Cally et al., 2019; Whitlock & Agrawal, 2009; Winkler et al., 2021). The role of good genes as a target for mate preferences remains controversial however (Achorn & Rosenthal, 2020; Rosenthal, 2017). This is perhaps in part because we are often studying, in the field or the lab, populations that are not currently undergoing strong selection (e.g. conditions amenable for fieldwork, or kept under benign conditions or free of parasites; in effect introducing genotype by environment interactions for fitness: Hunt & Hosken, 2014; Ingleby et al., 2010), and so there is little additive genetic variation in fitness expressed across the genome for the populations being studied.

Alternatively, phenotypic condition, and hence sexual attractiveness or competitiveness, may reflect the environmental circumstances an individual experiences. Individuals – regardless of genotype – that have faced environmental stress, including during development, may be less able to (i) avoid predators or pathogens, (ii) provide parental care or (iii) provide as high a quantity or quality of gametes, than individuals in better phenotypic condition. There is now a large literature suggesting that sexually selected traits are condition‐dependent, often responding to phenotypic manipulations of condition. Examples range from classic studies on house finches (*Haemorhous mexicanus*: Hill, 1990) and stalk‐eyed flies (*Cyrtodiopsis dalmanni*: Cotton et al., 2004a, 2004b), to recent work on sexual communication in túngara frogs (*Engystomops pustulosus*: Wilhite & Ryan, 2024) and male genitalia in the fruit fly *Drosophila simulans* (Pennell et al., 2024; for reviews see Dougherty, 2021; Hill, 2011; Johnstone et al., 2009). Importantly, it is not only male ornaments that can be condition‐dependent, so too can female ornaments (Hernández et al., 2021). Mate preferences can likewise be manipulated via changes in condition (Hunt et al., 2005). Interestingly, poor condition may either reduce or increase mate preferences (Fisher & Rosenthal, 2006; Fox & Moya‐Laraño, 2009), or indeed have no effect at all (Asakura et al., 2024). However, the evidence suggests that generally such effects are rather small (Dougherty, 2023).

Condition will therefore likely comprise genetic and environmental components, and their interactions. More complicated still is defining what ‘condition’ or ‘quality’ actually means, and then how to measure it. Empirically, relationships between factors such as size and weight have often been used to estimate ‘condition’, but ultimately the only useful guide to what constitutes ‘good condition’ is how the measure maps (positively) to fitness (for discussions see Hunt et al., 2004; Kotiaho, 1999, 2001, 2002; Wilson & Nussey, 2010).

Here, we consider the role of condition in pre‐ and post‐copulatory sexual selection in the seed bug *Lygaeus simulans*. Both males and females of this species mate multiply. In terms of sexual selection, evidence to date suggests that generally larger males and females are more likely to engage in a copulation (pre‐copulatory sexual selection; though note the male effect is less consistent than the female effect in both *L*. *simulans* and its sister species *L*. *equestris*: Balfour et al., 2020a, 2024; Dougherty et al., 2015; Dougherty & Shuker, 2016). The strongest and most consistent pattern of all is that larger females are more likely to receive sperm than smaller females, leading to higher levels of mating failure (copulations which result in no offspring: Greenway et al., 2015) in smaller females. This suggests cryptic male choice for large and more fecund females (post‐copulatory sexual selection: Balfour et al., 2020a, 2024; Dougherty & Shuker, 2014, 2016). This failure for females to receive sperm is non‐trivial, as mating failure rates in both *L. simulans* and *L*. *equestris* typically reach 40%–60% of copulations (Balfour et al., 2020a; Greenway et al., 2017; Greenway & Shuker, 2015; Micholitsch et al., 2000; Tadler, 1999; Tadler et al., 1999). What is perhaps strange in this system is that post‐copulatory mate choice seems to be strong, while pre‐copulatory choice – by both males and females – seems weaker, with even heterospecific matings – including across genera – occurring relatively frequently, suggesting limited pre‐copulatory assessment (Balfour et al., 2020b; Burdfield‐Steel & Shuker, 2014a; Shuker et al., 2015).

To explore further patterns of pre‐ and post‐copulatory sexual selection in *L*. *simulans* and go beyond body size as a potential indicator of mate quality, we manipulated body condition while experimentally limiting body size variation in both males and females. As a preliminary experiment, we first assessed how long adult bugs could survive without access to food and water. From this, we decided that short‐term limitation of access to water was an experimentally tractable, and biological relevant, variable to manipulate to influence body condition. Lygaeids are generally lacerate‐flush feeders, requiring saliva delivery to enable feeding, and so water is an important resource for feeding as well as metabolic maintenance (Burdfield‐Steel & Shuker, 2014b). We then asked whether access to water influences mate preferences and post‐copulatory sexual selection. If males prefer females in good condition, then we predict that males would be more likely to copulate with females that had continued access to water compared to females that had been water deprived. Likewise, females may also prefer males in good hydration condition (Edvardsson, 2007; Ivy et al., 1999), as we expect that this will enable them to provide more complete ejaculates than males limited in their water resources, or indeed the ejaculate itself might be a useful source of water, especially for water‐deprived females. How condition of the male or female chooser in turn influences preferences is perhaps harder to predict a priori, not least as effects may be small (Dougherty, 2023). However, a partner in better condition may be more crucial if the chooser is in poorer condition, which might be more important for poorly hydrated females for example. We would then expect individuals in poor condition to prefer more strongly a partner in good condition and thus would expect an interaction between female and male hydration treatments.

## 2 MATERIALS AND METHODS

### 2.1 Ethics statement

All the work described here complied with local and national animal welfare regulations. We used the insect *Lygaeus simulans* for which no review is necessary for carrying out experiments. There are no welfare or environmental implications of the experimental design or procedures.

### 2.2 Husbandry

*Lygaeus simulans* adults were collected from a population in Tuscany, Italy, in 2008 and 2009. Bugs are maintained in continuous culture population boxes (30 × 15 × 15 cm plastic boxes) and provided with approximately 500 g of de‐husked organic sunflower seeds, 2–3 cotton‐bunged tubes of deionised water (25 mL) and cotton wool for shelter. There are at least two population boxes maintained at any one time, with more boxes generated as experiments required. Water tubes are changed weekly and the population boxes are kept in an incubator (LMS 600/1200 Series 4 cooled incubators) at 29°C on a 22:2 h light: dark cycle to prevent the onset of reproductive diapause. New population boxes are created approximately every 6–10 weeks by transferring around 50 adults and nymphs across each instar to a new box. When setting up a new box, individuals are taken from multiple separate population boxes to prevent inbreeding.

Our experiment aimed to manipulate body condition to see how condition affected patterns of pre‐and post‐copulatory sexual selection. To identify an appropriate and experimentally tractable manipulation, we first tested the effects of food and/or water deprivation to work out the best way of providing a simple manipulation of adult body condition. The details of this preliminary experiment, including methods and results, are given in the Appendix. The results of this preliminary work suggested that short‐term deprivation of water was a suitable way to manipulate condition and is also relevant for the feeding ecology of this species (see Section 1). We therefore tested whether water‐deprivation for 24 h prior to a copulation opportunity influenced pre‐ and post‐copulatory sexual selection for both females and males.

The bugs used in the experiment were of the pale mutant colour morph (described in Balfour et al., 2018). To obtain virgin males and females for the experiment, we moved late instar nymphs to nymph boxes (20 × 10 × 8 cm plastic boxes) and provided them with an ad libitum supply of sunflower seeds, a cotton‐bunged tube of deionised water (25 mL) which was changed weekly, along with cotton wool for shelter. Nymph boxes were checked every 2–3 days for newly eclosed adults. Adults were transferred to collection tubs (108 × 82 × 55 mm plastic deli tubs) using storksbill forceps and were separated by sex. No more than 10 individuals were housed in a collection tub, where they were again provided with an ad libitum supply of sunflower seeds, a cotton‐bunged tube of deionised water (7 mL) and a piece of cotton wool. All boxes and tubs were kept in the incubator as for population boxes.

### 2.3 Experimental protocol

To focus on condition rather than body size, we isolated virgin males and females that fell into an intermediate (or ‘medium’) size class. This was to try and minimise size effects on the results, for example, since there is evidence for both pre‐ and post‐copulatory selection for larger females (see Section 1). We measured males and females by gently placing them between two glass slides to hold the bug still. We used a calibrated dissecting microscope fitted with an eyepiece micrometre, and the body length from the end of the snout to the tip of the wings was measured. Bugs in the medium category were the mean length of individuals of a given sex ± half a standard deviation (SD). The means and SDs of males and females were taken from Balfour et al. (2018). Medium females ranged in body length from 11.4 to 11.8 mm in length, while males ranged from 10.5 to 10.9 mm.

For 24 h prior to the experimental trials, bugs were subjected to one of two treatments. We randomly selected half of the tubs of measured females, and half of the tubs of measured males, and then removed their water tubes before returning them to the incubator until experimental trials commenced the following morning. The other half of the tubs did not have their water tubes removed, however, all tubs were removed from the incubator and handled regardless of whether the water tube was removed or not to control for the disturbance of the manipulation.

There were four treatment combinations in a 2 × 2 factorial design. The treatment codes were as follows: BB, BX, XB, XX, with the first letter denoting the condition of the female, the second letter the condition of the male, with B = had both water and food in the 24 h prior to the experiment, and X = was deprived of water 24 h prior to the experiment. The sample sizes were *N* = 65, 64, 78, 84, respectively. After the removal of seven data points due to missing data, bugs deaths during trials, and one pair which subsequently produced nymphs but which were not observed mating (possibly due to accidental transfer of an egg on forceps from another tub), the final sample sizes were *N* = 61, 63, 76, 82, respectively.

Mating trials were run for 6.25 h (instead of 6 h due to an error on the first experimental day, though we remained consistent with 6.25 h throughout the whole experiment), with pairs being observed every 15 min for copulation (with males and females in the classic back‐to‐back position), as is standard for our studies of sexual selection in this species (e.g. Balfour et al., 2024). These checks are frequent enough to capture all copulations, since 30 min is the minimum length of time required for sperm transfer to occur (Gschwentner & Tadler, 2000). Moreover, since copulations can last up to 24 h in the sibling species *L. equestris* (Kugelberg, 1973; Sillén‐Tullberg, 1981), it was not feasible for observations to continue for this length of time. Therefore, any pairs still *in copula* at the end of the trial were separated by gently brushing the genitalia with a paintbrush. Females were placed into individual tubs at the end of the trial, or after pairs broke apart after copulating for 3 or more consecutive checks, as we wanted to ensure only one successful copulation had occurred. The tubs were provisioned with 20–30 sunflower seeds and a water tube (7 mL). These females were then returned to the incubator for 7 days to lay eggs (note that females can lay unfertilised eggs, even if they have not copulated). We then euthanised the females by placing them in the freezer at −18°C and counted all the eggs present in each tub. Tubs with eggs were returned to the incubator for a further 7 days after which they were frozen for a minimum of 24 h at −18°C and we counted any nymphs present.

### 2.4 Statistical analysis

All statistical analyses were carried out using R version 3.6.1 (R Core Team, 2019). We used Generalised Linear Models (GLMs) with a binomial distribution and logit link function to test the effect of female and male condition (i.e. water deprived or not) and an interaction between these treatments on (i) the likelihood of a pair copulating, (ii) the likelihood of a female laying eggs, (iii) the likelihood of a pair producing nymphs (i.e. mating failure; see Section 1) and (iv) whether pairs initiated copulation within the first 15 min of the experimental trial. We also used a binomial GLM to investigate (v) the relationship between female condition, male condition and copulation duration on the likelihood of mating failure. We consider measures associated with pre‐copulatory sexual selection to be (i) and (iv), while (ii), (iii) and (v) are associated with post‐copulatory sexual selection. Additionally, we ran a Gaussian GLM to test the effect of bug condition on the number of eggs laid, and a quasibinomial GLM (to account for overdispersion) to test the effect of bug condition on the proportion of eggs which hatched. These also represent post‐copulatory outcomes. We used ‘Type II sums‐of‐squares’ throughout when testing main effects and interactions, with ‘*F*’ tests for the Gaussian GLMs and quasibinomial GLMs, and likelihood‐ratio (‘LR’) tests for the binomial GLMs, presented as χ^2^ test statistics. Note that for the statistical analysis, pairs were considered to have copulated if they were observed in copula for three or more consecutive checks (>30 min) during the mating trials, as this is the minimum length of time required for sperm transfer (Gschwentner & Tadler, 2000). Any pairs observed in copula for two or fewer consecutive checks were recorded as not having copulated. This is in line with previous experiments and analysis done on this species.

## 3 RESULTS

Of the 282 pairs used in this experiment, 196 of these engaged in copulation (69.5%) and 122 of these resulted in offspring, giving an overall mating failure rate of 37.8%.

**FIGURE 1:**
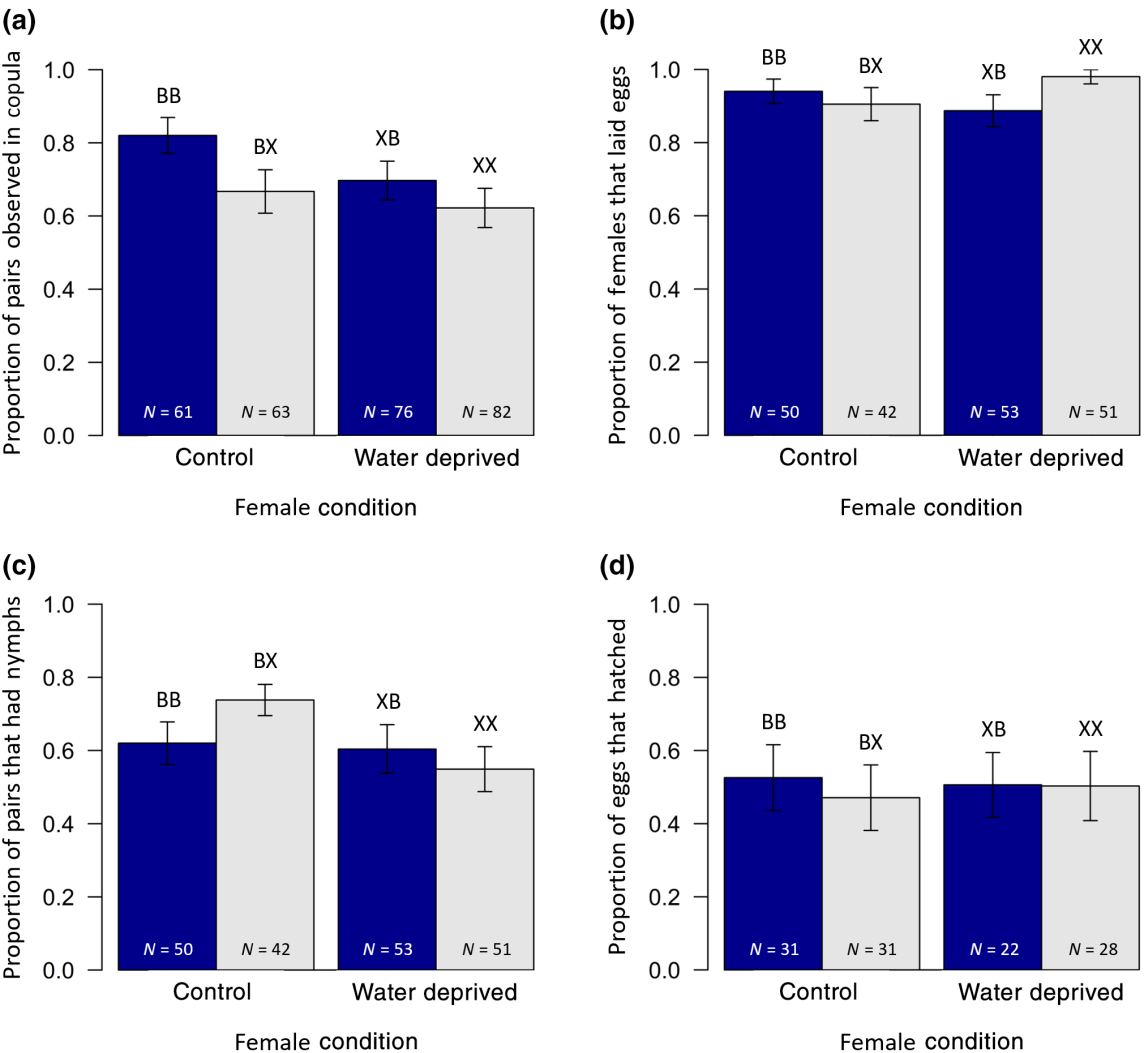
The proportion of (a) pairs observed in copula, (b) females observed in copula which subsequently laid eggs, (c) pairs which were observed in copula and successfully produced nymphs and (d) eggs that hatched (from pairs which produced nymphs), with respect to female condition. Solid blue bars represent pairs in which the male was not water deprived prior to the experimental trial (control), grey bars the pairs in which the male was water deprived. Error bars represent the standard error. Treatment codes are shown above each bar (first letter = female condition; second letter = male condition; B = control, not water deprived prior to experimental trial; X = water deprived 24 h prior to experimental trial). Sample sizes are shown on each bar.

In terms of pre‐copulatory sexual selection, males in good condition were slightly more successful in copulating than water‐deprived males in poor condition ($\chi_{1}^{2}$ = 4.04, *p* = .044; Figure 1a). Female condition, however, did not have an effect on the likelihood of a copulation occurring ($\chi_{1}^{2}$ = 2.28, *p* = .131), nor was there an interaction between male and female condition on the likelihood of engaging in a copulation (interaction: $\chi_{1}^{2}$ = 0.80, *p* = .372).

Of the pairs which mated, 55.6% did so within the first 15 min of the experimental trial. Whether pairs engaged in copulation in the first 15 min was influenced by condition, with a significant interaction between male and female condition (interaction: $\chi_{1}^{2}$ = 6.87, *p* = .009). This was because pairs in the treatment BB engaged in copulation much more quickly (76% within first 15 min) than pairs in any other treatment (BX = 48%; XB = 45%; XX = 53%). Put another way, males in good condition were far more likely to copulate with a good condition female in the first 15 min than a poor condition female, whereas poor condition males were as likely to copulate quickly with a female of either condition. The main effects of both male ($\chi_{1}^{2}$ = 1.71, *p* = .192) and female condition ($\chi_{1}^{2}$ = 3.76, *p* = .053) were not significant, although marginal in the latter case, with water‐deprived females perhaps tending to take longer to engage in copulation.

**FIGURE 2:**
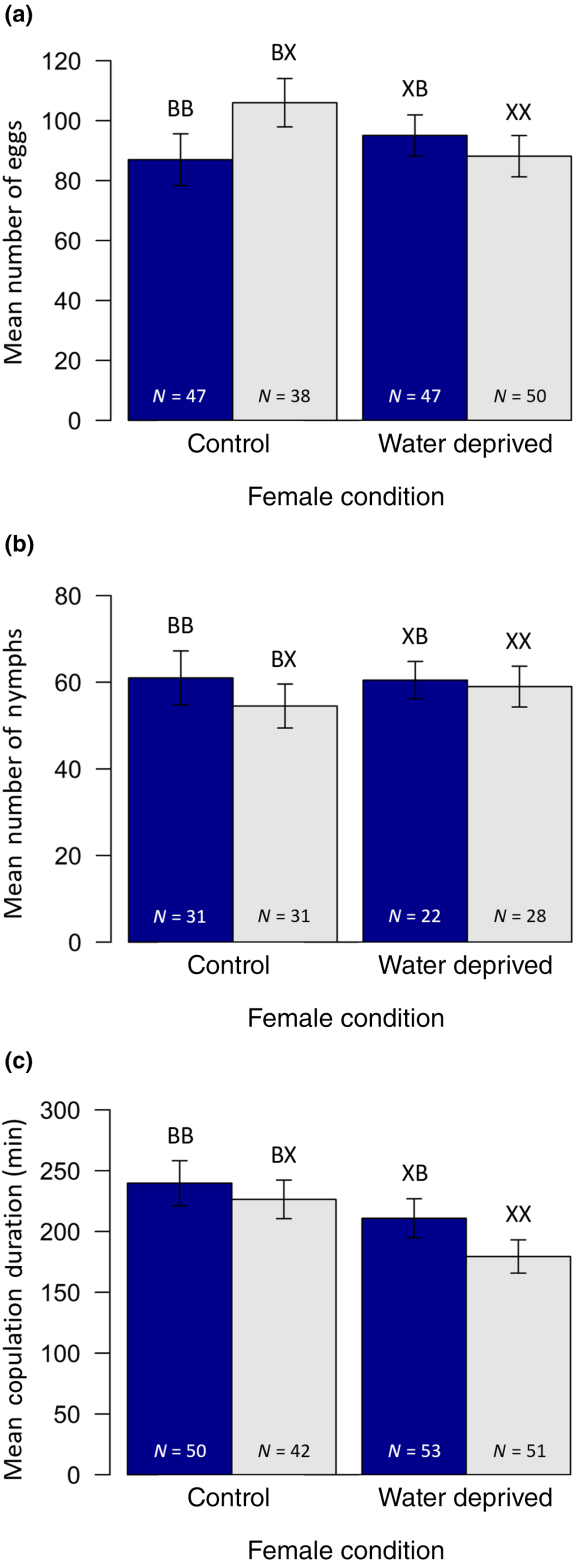
(a) Mean number of eggs laid by females which mated and laid eggs, (b) mean number of nymphs produced by pairs which had nymphs (and hence did not experience mating failure) and (c) mean copulation duration for pairs that copulated, depending on female condition. Solid blue bars represent pairs in which the male was not water deprived prior to the experimental trial (control), grey bars the pairs in which the male was water deprived. Error bars represent the standard error. Treatment codes are as given in Figure 1.

In terms of post‐copulatory sexual selection, females that copulated were significantly more likely to lay eggs (92.9%) than females that did not copulate (81.4%: $\chi_{1}^{2}$ = 7.63, *p* = .006). For females which copulated there was no effect of female condition ($\chi_{1}^{2}$ = 0.04, *p* = .836), nor male condition ($\chi_{1}^{2}$ = 0.83, *p* = .361), on the likelihood of laying eggs. However, there was a marginally non‐significant interaction between the two (interaction: $\chi_{1}^{2}$ = 3.59, *p* = .058; Figure 1b). Water‐deprived females tended to be more likely to lay eggs if they copulated with a water‐deprived male than a non‐water‐deprived male, whereas females that had access to water were as likely to lay eggs independent of male condition. Additionally, for females that copulated and laid eggs there was no effect of female condition (*F*_1,178_ = 0.32, *p* = .574), male condition (*F*_1,178_ = 0.45, *p* = .502), nor an interaction between the two (interaction: *F*_1,178_ = 2.87, *p* = .092), on the number of eggs laid (Figure 2a).

For pairs which copulated, the likelihood of experiencing mating failure did not depend on female condition ($\chi_{1}^{2}$ = 2.00, *p* = .158), male condition ($\chi_{1}^{2}$ = 0.14, *p* = .706) nor again any interaction between them (interaction: $\chi_{1}^{2}$ = 1.64, *p* = .200; Figure 1c). Likewise, for pairs which did not experience mating failure and so produced nymphs, the mean number of nymphs did not differ depending on female condition (*F*_1,118_ = 0.14, *p* = .712), male condition (*F*_1,118_ = 0.61, *p* = .435), nor any interaction (interaction: *F*_1,118_ = 0.23, *p* = .630; Figure 2b). For these pairs, the proportion of eggs that hatched also did not differ with respect to female condition (*F*_1,118_ = 0.02, *p* = .877), male condition (*F*_1,118_ = 0.83, *p* = .364) or the interaction (interaction: *F*_1,118_ = 0.66, *p* = .419; Figure 1d).

**FIGURE 3:**
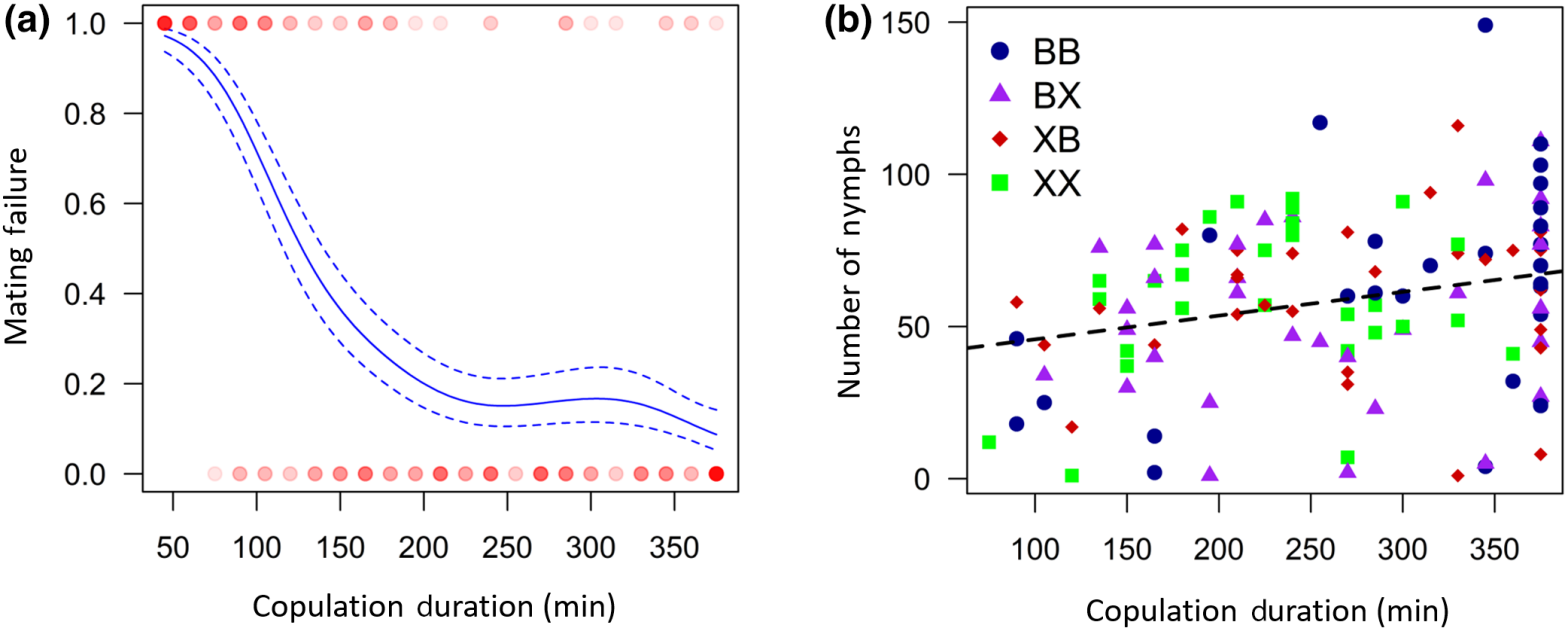
(a) Relationship between copulation duration and the rate of mating failure (whether pairs had nymphs [0] or not [1]), for pairs which mated, visualised as cubic splines (*N* = 196). Data are represented by circles, with the colour reflecting the number of individuals of the given size and mated state (darker = more replicates). Dashed lines indicate 1 standard error above and below the predicted line. (b) Relationship between copulation duration and the number of nymphs produced by pairs which successfully had nymphs, for each of the four treatments. A linear regression for all treatments combined is shown (dashed line; *N* = 122).

Turning to copulation duration, there was an effect of female condition on copulation duration (*F*_1,192_ = 5.34, *p* = .022) with pairings involving control females lasting longer than those with water‐deprived females. There was no effect of male condition though, (*F*_1,192_ = 2.01, *p* = .158), and no interaction between male and female condition (interaction: *F*_1,192_ = 0.31, *p* = .576; Figure 2c). The likelihood of mating failure occurring was strongly driven by copulation duration, with longer copulations being more likely to result in offspring production (*F*_1,194_ = 106.51, *p* < .001; Figure 3a). Furthermore, for pairs which produced offspring, the number of nymphs sired also increased with increasing copulation duration (*F*_1,120_ = 8.10, *p* = .005; Figure 3b).

## 4 DISCUSSION

Variation in body condition, whether environmental or genetic in origin, can play an important role in mate choice and hence sexual selection (Cotton et al., 2006). Here we have tested whether short‐term water deprivation influences patterns of male and female mate choice in the seed bug *Lygaeus simulans*. In terms of pre‐copulatory sexual selection, we found that whether pairs copulated or not depended marginally on male condition (but not female condition), with water‐deprived males being less likely to mate. In terms of post‐copulatory sexual selection, copulations with water‐deprived females were shorter in duration.

Both of these effects suggest that mating outcomes, and possibly mate choice, were influenced by condition. Poor condition males were selected against in terms of obtaining a mating. This could in part be due to males in less good condition being less able to coerce females into mating, with mate choice here resulting from female resistance to male coercion attempts (i.e. sexual conflict over mating: Arnqvist & Rowe, 2005; see Shuker et al., 2006 for a discussion of sexual conflict over mating in the closely related *Lygaeus equestris*). Females in poor condition were selected against in terms of copulation duration, which is in turn a key determinant of mating failure and offspring production (Balfour et al., 2020a, 2024). Indeed, these results add to the case that cryptic male choice is an important driver of female insemination success in this species (see below).

Certainly, there is evidence that both diet and body size can influence ejaculate size and composition in males (e.g. in the eastern mosquitofish *Gambusia holbrooki*: O'Dea et al., 2014; in the two‐spot ladybird *Adalia bipunctata*: Perry & Rowe, 2010; see also the review by Meuti & Short, 2019). Therefore, it seems likely that water should as well. If continuous access to water is necessary for the production of ejaculate components, including of course seminal fluid itself, then we might expect these males to be less willing or able to mate. Alternatively, it could be a case of female choice where females are less willing to copulate with poor condition males. In the seed beetle *Callosobruchus maculatus*, females appear to remate to gain the benefits of getting water from male ejaculates, as females deprived of water were more likely to remate than those that were not (Edvardsson, 2007; Harano, 2012; Ursprung et al., 2009). However, as we saw here, female *L. simulans* deprived of water were no more likely to copulate than those females which had access to water, so this may not be the case in this species. Moreover, male condition had no effect on post‐copulatory success, therefore the males which did copulate must have had sufficient ejaculate to inseminate females, as there was no difference in the likelihood of mating failure occurring, or the number of offspring produced, between the water deprived and non‐water deprived males which copulated.

Female body condition also had no influence on mating failure. However, copulations with females deprived of water were shorter than those involving females which did have access to water prior to the mating trial. If it is the case that males are in control of copulation duration, then this is more evidence towards cryptic male choice in this species (Balfour et al., 2024). Water availability can affect fecundity and egg production in other species (e.g. navel orangeworm *Amyelois transitella*: Burks, 2014; almond moth *Ephestia cautella*: Ryne et al., 2004) and so males may choose to copulate for shorter durations and transfer fewer sperm to females deprived of water. However, beyond the effects of copulation duration, water‐deprived females were not drastically hampered in at least their immediate fecundity. Our short‐term manipulation perhaps has little immediate effect (e.g. mature eggs will be ready to be oviposited), with effects on fecundity—if present—only playing out later.

As such, it is certainly fair to ask then to what extent have we successfully manipulated male and female condition? Prolonged (>48 h) deprivation of water has clear deleterious effects for the bugs (see Appendix) while food deprivation has much less of an effect. While some aspects of mating have been influenced by the manipulation, we did not see large, immediate differences in how males and females behaved, or a drastic drop‐off in reproduction. We were only looking at a very short window for responses though. For now, perhaps the most we can safely say is that short‐term water availability can influence whether copulations take place, and also copulation duration, an important component of mating success in this species, associated with mating failure in particular. Looking more broadly, there is now growing evidence that how the environment shapes fertility, including in terms of thermal limits for fertility, are likely to be far more crucial for species distributions than thermal survival tolerances (Bretman et al., 2024; Dougherty et al., 2024; Parratt et al., 2021; Van Heerwaarden & Sgrò, 2021; Walsh et al., 2019). Our data here suggest that short‐term water stress, associated with temperature or disrupted weather patterns, may likewise influence fertility in this species. However, further investigations are needed to get a clearer picture of if and how body condition influences sexual selection in this species. Recent developments in artificial diets for related species may offer a way forward (Espinosa del Alba & Petschenka, 2023), allowing us to manipulate both diet and water to explore how condition shapes reproduction in lygaeid bugs. With such manipulations we may also be able to extend our consideration of sexual selection and condition to include other aspects of sexual selection not considered here (Andersson, 1994), including scramble and contest competition, and endurance rivalry.

## AUTHOR CONTRIBUTIONS

**Vicki L. Balfour:** Conceptualization (lead); data curation (equal); formal analysis (lead); methodology (lead); supervision (supporting); writing – original draft (equal); writing – review and editing (equal). **Mia K. Corliss:** Data curation (equal). **David M. Shuker:** Conceptualization (supporting); funding acquisition (lead); methodology (supporting); supervision (lead); writing – original draft (equal); writing – review and editing (equal).

## CONFLICT OF INTEREST STATEMENT

The authors declare no competing nor conflicting interests.

## Figures and Tables

**FIGURE A1:**
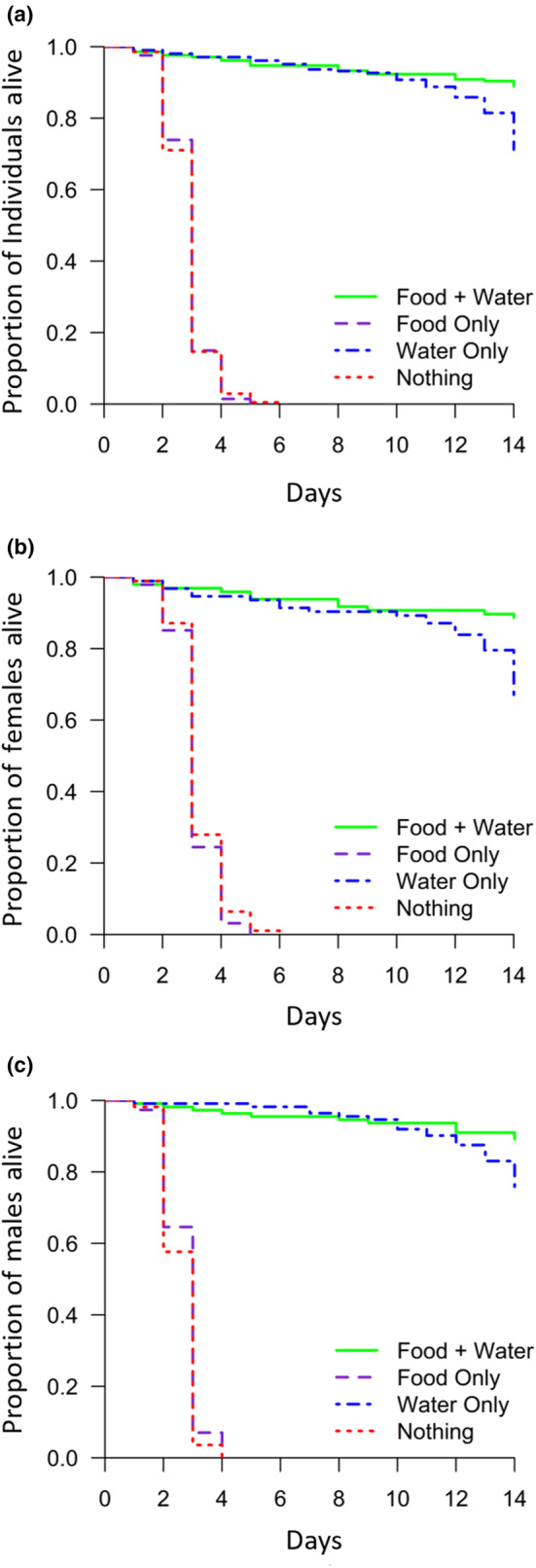
The proportion of (a) all, (b) female and (c) male *Lygaeus simulans* (including both pale and wild‐type phenotypes) alive over the course of 14 days in the preliminary experiment when housed individually and provided with (i) food and water, (ii) food only, (iii) water only and (iv) nothing. Note that the number of days is the number of days when kept in each treatment. All bugs were 7 days post‐eclosion when the experiment began. The respective sample sizes were (a) *N* = 208, 207, 205, 204, (b) *N* = 97, 94, 93, 93 and (c) *N* = 111, 113, 112, 111.

## Data Availability

The research data underpinning this publication can be accessed at https://doi.org/10.17630/2b19235c‐3e05‐4398‐b74e‐9dda08ff6840.

## 1

Here we provide the methods and results for the preliminary experiment to explore food and water deprivation to manipulate body condition in *Lygaeus simulans*. We wished to identify what food and water deprivation manipulations would result in short‐term changes but that would also avoid being overly deleterious and/or lethal.

For the preliminary experiment, we deviated from the standard husbandry protocol for collecting virgins. Instead of checking nymph boxes every 2–3 days, nymph boxes were checked daily for newly eclosed adults. This was to ensure we knew the exact age of the adults used. Adult *Lygaeus simulans* (7 days post‐eclosion) were placed in individual tubs (108 × 82 × 55 mm) and provided with either (i) 10 sunflower seeds and a cotton‐plugged Eppendorf tube filled with deionised water, (ii) only 10 sunflower seeds, (iii) only a cotton‐plugged Eppendorf tube filled with deionised water or (iv) nothing. The sample sizes were *N* = 208, 207, 205 and 204 respectively. We used both wild‐type and pale mutant *L*. *simulans* for the experiment (see Balfour et al., 2018 for further details of the pale mutant). The phenotype (pale or wild‐type) and the sex of the bugs were recorded. Bugs were checked daily for 2 weeks and any deaths and the death date recorded. When a bug died, or when the 2 week time period was up, the bugs were placed in individual Eppendorf tubes and frozen at −18°C so that their body length could be measured at a later date. We measured the body length (dorsal side up, from tip of the snout to end of the wings) of all the bugs after thawing from the freezer using a calibrated dissecting microscope fitted with an eyepiece micrometre.

We used the *survival* and *survminer* packages in R (Kassambara et al., 2021; R Core Team, 2019; Therneau, 2015) to analyse the data set. We used the Cox Proportional‐Hazards Regression model to test which variables affected the likelihood of survival. We note that the data may have partially violated some of the assumptions of the proportional hazards model because bugs without water died off very quickly at around the same time in the first few days, rather than consistently over time. Therefore, the results here must be treated with caution, however the statistical analysis matches what we see in the data, and more complicated modelling does not seem necessary (Tables A1 and A2; Figure A1). In the model, we included the body length, sex, whether bugs had water or not, whether bugs had food or not, and phenotype. We also included an interaction between sex and access to water, in addition to an interaction between water access and food access. There was no effect of body length (*z* = −0.662, *p* = .508) or phenotype (*z* = −0.569, *p* = .570) on survival. However, there was a highly significant effect of whether bugs had water or not, with all bugs dying after 5 days without water (*z* = −13.27, *p* < .001). There was also a significant effect of sex (*z* = 4.990, *p* < .001), and there was an interaction between sex and presence of water (interaction: *z* = −4.031, *p* < .001). The majority of males died after 2–3 days of having no water, whereas the majority of females died after 3–4 days of having no water (Table A1). No males survived more than 3 days without water, whereas no females survived more than 5 days without water (Table A2).

For bugs which had water, there was no difference in survival between the sexes (Log‐Rank Test: $\chi_{1}^{2}$ = 1.7, *p* = .200), however, males died faster than females when deprived of water ($\chi_{1}^{2}$ = 54.4, *p* < .001; Figure A1b,c). On its own, whether bugs had food or not was not significant (*z* = −0.295, *p* = .768), however, there was an interaction between presence of food and presence of water (interaction: *z* = −4.137, *p* < .001). Exploring this further using Log‐rank tests, when comparing only bugs which did not have water, there was no difference in survival between bugs which did or did not have food ($\chi_{1}^{2}$ = 0, *p* = .9). However, for bugs which did have access to water, more bugs died when deprived of food ($\chi_{1}^{2}$ = 18.4, *p* < .001). Looking at Figure A1a we can see this trend occurs after 10 days without food.

**TABLE A1:** The number of individuals which died after 1, 2, 3, 4, 5 or 6 days without water in the preliminary experiment, with respect to sex and phenotype. Note that the numbers given are those died per day, not the cumulative number of deaths.

Sex	Phenotype	*N*	No. individuals that died after X No. days without water					
			1	2	3	4	5	6
Male	Pale	114	3	44	59	8	0	0
	Wild‐type	110	2	38	66	4	0	0
	Combined	224	5	82	125	12	0	0
Female	Pale	90	1	10	62	15	2	0
	Wild‐type	97	2	13	50	25	6	1
	Combined	187	3	23	112	40	8	1

**TABLE A2:** The proportion of individuals left alive after 1, 2, 3, 4, 5 or 6 days without water in the preliminary experiment with respect to sex and phenotype.

Sex	Phenotype	*N*	Proportion of individuals alive after X No. days without water					
			1	2	3	4	5	6
Male	Pale	114	0.97	0.59	0.07	0.00	0.00	0.00
	Wild‐type	110	0.98	0.64	0.04	0.00	0.00	0.00
	Combined	224	0.98	0.61	0.05	0.00	0.00	0.00
Female	Pale	90	0.99	0.88	0.19	0.02	0.00	0.00
	Wild‐type	97	0.98	0.85	0.33	0.07	0.01	0.00
	Combined	187	0.98	0.86	0.26	0.05	0.01	0.00
